# NF-κB Mediates the Expression of *TBX15* in Cancer Cells

**DOI:** 10.1371/journal.pone.0157761

**Published:** 2016-06-21

**Authors:** Jéssica Arribas, Tatiana Cajuso, Angela Rodio, Ricard Marcos, Antonio Leonardi, Antonia Velázquez

**Affiliations:** 1 Grup de Mutagènesi, Unitat de Genètica, Departament de Genètica i de Microbiologia, Facultat de Biociències, Universitat Autònoma de Barcelona, Cerdanyola del Vallés, Barcelona, Spain; 2 CIBER Epidemiologia y Salud Pública, Instituto de Salud Carlos III (SCIII), Madrid, Spain; 3 Dipartimento di Biologia e Patologia Cellulare e Molecolare, Università Federico II, Napoli, Italy; University of Navarra, SPAIN

## Abstract

TBX15 is a T-box transcription factor essential for development, also proposed as a marker in prostate cancer; and, recently, its antiapoptotic function indicates a role in carcinogenesis. Regulation of *TBX15* is uncovered. In this study, we investigated the regulation of *TBX15* expression in human cancer cells, by analyzing the regulatory function of a 5’-distal conserved region of *TBX15*. Bisulfite sequencing showed high methylation of the CpG island contained in this region that was not correlated with *TBX15* mRNA levels, in the cancer cell lines analyzed; however, after 5-aza-dC treatment of TPC-1 cells an increase of *TBX15* expression was observed. We also found a significant response of *TBX15* to TNF-α activation of the NF-κB pathway using five cancer cell lines, and similar results were obtained when NF-κB was activated with PMA/ionomycin. Next, by luciferase reporter assays, we identified the *TBX15* regulatory region containing two functional NF-κB binding sites with response to NF-κBp65, mapping on the -3302 and -3059 positions of the *TBX15* gene. Moreover, a direct interaction of NF-κBp65 with one of the two NF-κB binding sites was indicated by ChIP assays. In summary, we provide novel data showing that NF-κB signaling up-regulates *TBX15* expression in cancer cells. Furthermore, the link between *TBX15* and NF-κB found in this study may be important to understand cancer and development processes.

## Introduction

*TBX15* is a member of the conserved T-box gene family that is essential for many developmental processes [1]. *TBX15* encodes a transcription factor involved in the development of the skeleton [2]. In humans, the Cousin syndrome is characterized by craniofacial and skeletal defects with scapular and pelvic hipoplasia and is caused by *TBX15* mutations [3, 4]. The *Tbx15*-deficient mouse, droopy ear, and engineered Tbx15-null mutants presented similar phenotype than the Cousin syndrome in addition to pigment pattern alterations [5, 6, 7]. More recently, it has been reported that *TBX15* is involved in adipocyte differentiation and mitochondrial respiration [8, 9]. Also, accumulating evidences indicate a role of T-box genes in differentiation, proliferation and apoptosis, which are relevant processes in carcinogenesis [10, 11], in addition to the altered expression of diverse T-box genes associated with a variety of cancers [12].

The information about *TBX15* expression is mainly based on developmental studies, *TBX15* being expressed in the mesenchyme during limb development, in mesenchymal precursor cells, chondrocytes and skeletal musculature [2]. Also, differentiation of the distinct adipocyte lineages showed differential expression of *TBX15* [9]. To date, the regulatory mechanisms of *TBX15* remain poorly understood, but considering the dynamic and temporal expression of *TBX15* regulatory systems that are tissue and stage specific can be anticipated. There are also some hints suggesting that both genetic and epigenetic factors would regulate *TBX15* transcription. One study carried out in human placentas, indicated that PDX1 regulates *TBX15* in a methylation-dependent manner [13]; and methylation of *TBX15* has also been proposed as a prognostic marker for prostate cancer [14].

Recently we found that *TBX15* has an antiapoptotic role and its expression was altered in cancer cells [15]. These results are correlated with our previous observation that a region in chromosome 1p12 containing the *TBX15* gene was associated with thyroid cancer susceptibility in humans [16]. Thus, to understand the implication of *TBX15* on cell proliferation and survival related to carcinogenesis is important, and it requires uncovering the regulation of *TBX15* expression. In this study, we investigate the regulatory function of a highly conserved region in the distal promoter of *TBX15*. This region extends the CG rich regulatory sequence of *TBX15* found in human placentas by Chelbi *et al*. [13]. We identified a NF-κB responsive region with a positive effect in the transcription of *TBX15* in human cancer cells.

## Material and Methods

### Cell culture

The human cell lines used for this study were from papillary thyroid cancer: TPC-1 and BCPAP; from follicular thyroid cancer: WRO and CGTH; from anaplastic thyroid cancer: FRO, 8305, BHT-101 and BHT-101 IκBαSR; from cervical cancer: HeLa; and the human embryonic kidney cell line HEK293. The TPC-1 and BHT-101 cell lines were provided by Dr. R. Melillo and Dr. M. Santoro (Università Degli Studi Di Napoli, Naples, Italy), and the WRO and FRO cells by Dr. R. Ciampi (University of Pisa, Pisa, Italy). The HeLa cell line was obtained from the Centro Nacional de Investigaciones Oncológicas (CNIO, Madrid, Spain) and the HEK293 was provided by Dr. F. Rosselli (Gustave Roussy, Paris, France). BHT-101 IκBαSR (constitutively express an IκBα super-repressor) cell line has been generated by infecting BHT-101 WT cells with a lentivirus encoding a super-repressor form of IkBα. All laboratories guaranteed the identity of these cell lines, providing the cell lines with a low number of passages. The human thyroid cancer cell lines BCPAP, CGTH and 8305C were purchased from the Leibniz-Institut DSMZ—Deutsche Sammlung von Mikroorganismen und Zellkulturen GmbH cell bank (DSMZ). The DSMZ assured cell lines identity by regular analysis using the authentication method based in short tandem repeat DNA fingerprint. The TPC-1, HeLa and HEK293 cell lines were grown in DMEM medium (Sigma-Aldrich) containing 10% heat-inactivated FCS (Gibco^®^) and 2.5μg/mL of Plasmocin^™^ (InvivoGen). BHT-101 and BHT-101 IκBαSR cell lines were grown in DMEM medium (Sigma-Aldrich) containing 20% heat-inactivated FCS (Gibco^®^), glutamine and 2.5μg/mL of Plasmocin^™^ (InvivoGen). WRO and FRO cells were grown in RPMI 1640 medium (Sigma-Aldrich) supplemented with 10% heat-inactivated FCS (Gibco^®^), 1% no essential amino acids (Sigma-Aldrich), 1% sodium pyruvate (Sigma-Aldrich) and 2.5μg/mL of Plasmocin^™^ (InvivoGen). BCPAP, CGTH and 8305C cells were grown in RPMI 1640 medium (Sigma-Aldrich) containing 10% heat-inactivated FCS (Gibco^®^) and 2.5μg/mL of Plasmocin^™^ (InvivoGen).

The two mouse embryonic fibroblast cell lines, p65^+/+^MEF and p65^-/-^MEF, were kindly provided by Dr. G. Franzoso (The Gwen Knapp Center for Lupus and Immunology research, The University of Chicago, USA). These cells were grown in DMEM medium (Sigma-Aldrich) containing 10% heat-inactivated FCS (Gibco^®^) and 2.5 μg/mL of Plasmocin^™^ (InvivoGen).

Cells were placed in culture flasks and incubated in a humidified atmosphere (5% CO2) at 37°C. All experiments were performed at passages <20–25.

### Prediction of transcription factor binding sites

A DNA sequence of approximately 1kb in the 5-flanking region of the *TBX15* gene was obtained from Ensembl GRCh38 human genome database (http://www.ensembl.org/). Prediction of transcription factor binding sites was assessed using four online prediction softwares with a score cutoff > 85%, MatInspector (www.genomatix.de), rVista 2.0 (http://rvista.dcode.org), TFSEARCH (http://www.cbrc.jp/research/db/TFSEARCH.html) and CONSITE (http://consite.genereg.net/).

### DNA isolation and bisulfite sequencing

Phenol-chloroform total DNA extraction was performed from TPC-1, BCPAP, WRO, CGTH, FRO, 8305 and HeLa cell lines. Genomic DNA was modified with sodium bisulfite using the EZ DNA Methylation-Startup^™^ Kit (Zymo Research, USA) and following the manufacturer’s instructions. Using bisulfite-modified DNA, the genomic region covering the CpG island of the *TBX15* distal promoter (-3852/-3381 from the *TBX15* transcription start site) was amplified in two overlapping fragments (C1, 250bp and C2, 288bp) followed by sequence analysis. The primers sequences were designed using the DNA Methylation Analysis PCR Primer Database [17]. Each PCR reaction was performed in a total volume of 25μL containing 50–100 ng of bisulfite-modified DNA of each cell line, 1 x ZymoTaq^™^ PreMix (Zymo Research, USA) and 1mM of each primer. PCR reaction containing Universal Methilated Human DNA Standard (Zymo Research, USA) was carried out as control reaction. The amplification conditions were: 95°C for 10 min; 40 cycles of 95°C for 35s, annealing temperature (Ta) for 30s, and 72°C for 1min; and a final extension step for 7 min at 72°C. The primers and their annealing temperature (Ta) are listed in S1 Table. The amplified PCR products were separated by agarose gel electrophoresis, purified and sequenced. Methylation levels were estimated using the semiquantitative method described by Jiang *et al*., [18], applying the following formula: % Methylation = 100 X numberC/(numberC + numberT). Percentages were rounded off to the ranges: 0%, 25%, 50%, 75% and 100%.

### Genomic DNA demethylation

TPC-1 cells were seeded in 6-well plates at 10^5^ cells/well (roughly 20% of confluence). After 24h, the DNA demethylating reagent 5-aza-2′-deoxycytidine (5-aza-dC, Sigma-Aldrich) was added to the final concentrations of 2 μM or 5μM and the cells were growing for 96h. Fresh 5-aza-dC was replaced every 24 hours. On day 5, 5-aza-dC treated cells were harvested for RNA extraction and RT-qPCR analysis.

### TNFα and phorbol 12-myristate 13-acetate (PMA)/ionomycin (I) stimulation

TNFα (Peprotech) stock solution aliquots in culture medium were 10^6^ U/mL TNFα. FRO, CGTH, TPC-1, HeLa and MEF cells were seeded in 6-well plates (10^5^ cells/well). When cells were attached, culture medium was replaced with fresh medium containing 2000 U/mL TNFα as a final concentration, or fresh medium for control cultures. After 24h of TNFα treatment, cells were harvested for RNA extraction and RT-qPCR analysis.

BHT-101 and BHT-101 IκBαSR cells were seeded in 6-well plates (4x10^5^ cells/well). When cells were attached, culture medium was replaced with fresh medium containing 2000 U/mL TNFα, or 2 μM PMA (Sigma-Aldrich) plus 400 ng/mL I (Sigma-Aldrich) as a final concentration, or fresh medium for control cultures. After 3h of treatment, cells were harvested for RNA extraction and RT-qPCR analysis.

### RNA isolation and RT-qPCR analysis

Isolation of total RNA from cell samples was performed using TRIzol^®^ Reagent (Ambion^®^, Life Technologies^™^), following manufacturer’s protocol.

cDNA synthesis was carried out with 1 μg of total RNA using the Transcriptor First Strand cDNA Synthesis Kit (Roche). The quantitative expression of the *TBX15*, *CXCL1* and *RPL27* genes was analyzed by Real Time PCR using LightCycler^®^ 480 SYBRGreen I Master according to the manufacturer's protocol. Gene specific primers are listed in S1 Table. Each real-time PCR reaction was performed for 5 min at 95°C, 45 cycles at 95°C for 10s, 58°C for 15s, 72°C for 25s, and 78°C for 5s. The specificity of the reaction was verified by dissociation curve analysis using a cycle of 95°C for 5s followed by constant increase of temperature between 65°C and 95°C. Reactions were carried out in a LightCycler^®^ 480 System. Relative expression levels of the *TBX15* and CXCL1 genes were normalized to the housekeeping gene *RPL27* (internal control) and were calculated using the 2nd Derivative Max Method. Each real-time PCR reaction was performed in triplicate.

### Construction of the *TBX15* promoter reporter plasmids

Five different fragments of the 5-flanking region of the human *TBX15* gene were generated by PCR using the specific primers listed in S1 Table that included the restriction sites for BglII and KpnI (New England Biolabs) to direct cloning into the firefly luciferase vector pGL4.26 (Promega). PCR conditions were 95°C for 5 min followed by 35 cycles at 95°C for 45s, 65°C for 45s and 72°C for 1min 45s. The reporter constructs were: P1 (-3478/+206), P2 (-3478/-2865), P2rev (-2865/-3478), P3 (-3427/-2865) and P4 (-3587/-3283). The constructs were confirmed by restriction mapping and sequencing.

Site-directed mutants of the reporter construct P2 were generated using the QuickChange II XL Site-Directed Mutagenesis Kit (Agilent Technologies), following the manufacturer’s indications. The three putative NF-κB binding sites of the *TBX15* promoter contained in the construct P2 were mutated using primers with EcoRI, KpnI or kBS2 restriction sequences to substitute part of the kBS1, kBS2 or kBS3 sequences, respectively. The mutated P2 constructs comprised three NF-κB single mutated plasmids (P2mutS1, P2mutS2 and P2mutS3) and a NF-κB triple mutated plasmid (P2mutS1+S2+S3). Mutated reporter constructs were verified by sequencing.

### Luciferase reporter assays

HEK293 cells were seeded in 6-well plates (10^5^ cells/well) and transfected using Lipofectamine^®^ 2000 Transfection Reagent (Life Technologies), following the standard manufacturer’s protocol. Cells were transfected with 0.5 μg/well of the appropriate *TBX15* promoter reporter construct and 0.25μg/well of renilla luciferase reporter (Promega) as internal control for transfection efficiency. After 24h of TNFα stimulation, cells were lysated in PLB buffer, and subsequently assayed for luciferase activity. When the expression of p65 was required, cells were cotransfected with promoter reporter construct and the pCDNA3.p65 expression plasmid (kindly provided by Dr. Leonardi, Naples, Italy) by using 0.15μg/well of luciferase reporter construct, 0.05μg/well of renilla reporter and 0.8μg/well of pCDNA3.p65 (or empty pcDNA3.1 as negative control). After 24h, cells were lysated and assayed for luciferase activity. Luciferase activity was measured using the Dual-Luciferase^®^ Reporter Assay System (Promega). All transfections were performed in duplicate, and experiments were repeated at least three times. The firefly luciferase activity was normalized according to the renilla luciferase activity.

### Chromatin immunoprecipitation (ChIP)

The ChIP assay was as described by Nowak *et al*. [19] with modifications, using TPC and CGTH and solutions specified in S2 Table. Cells were seeded in plates at 5x10^6^ cells/plate with growth medium supplemented with 0.5% bovine serum albumin. After 24 h, cells were stimulated with 2000 U/mL TNFα for 30 min, washed at room temperature with PBS and after with PBS/Mg. TNFα stimulated cells were cross-linked with 2 mM disuccinimidyl glutarate (DSG, Fisher Scientific) for 45 min, washed with PBS, and fixed with 1% freshly prepared solution of formaldehyde in PBS/Mg (v/v) for 15 min at room temperature. Then fixed cells were washed with PBS, scraped, transferred into eppendorf tubes and lysed in 900 μL of L1 buffer for 15 min on ice. Nuclei were precipitated by centrifugation and suspended in 500 μL of SDS lysis buffer at room temperature. The samples were sonicated on a Digital Branson Sonifier^®^ (Branson Ultrasonics Danbury, USA) on ice during 7 min in cycles of 30s on/30s off. Low ionic strength dilution buffer was added to the soluble chromatin to a final volume of 900 μL with 20 μL of magnetic protein G beads (Protein G Mag Sepharose Xtra, GE Healthcare Life Sciences). p65 antibody-magnetic beads complexes were prepared by adding 4 μg of p65 antibody (NFKB p65 sc-372-G of Santa Cruz Biotechnology) to a final volume of 500 μL of low ionic strength dilution buffer with 20μL of magnetic beads, and overnight incubation at 4°C with rotation. Then washed with low ionic strength dilution buffer and added to sheared chromatin suspension and incubated with rotation at 4°C overnight. 10 μL of chromatin suspension were removed before immunoprecipitation to determine the imput quantity of DNA. Immunocomplexes were captured using a magnetic stand (MagRack 6, GE Healthcare Life Sciences), and washed twice with 500μL of low ionic strength dilution buffer, high-salt wash buffer, LiCl wash buffer, and twice with TE. Precipitated complexes were eluted with 400 μL of elution buffer for 30 min at room temperature with rotation. Samples were de-cross-linked in 200 mM NaCl, 50 mM Tris pH 6.8, 10mM EDTA adding 200 μg/mL proteinase K and incubated at 65°C for 2 h 30 min. DNA was extracted by phenol-chloroform, precipitated using ammonium acetate/ethanol and used for qPCR analysis. PCR primers and conditions for quantitative PCR are shown in S1 Table.

### Statistical analysis

The data were expressed as means ± standard deviations (SD) and analyzed by Student's t-test. p-value <0.05 was considered to indicate statistical significance.

## Results

**Fig 1:**
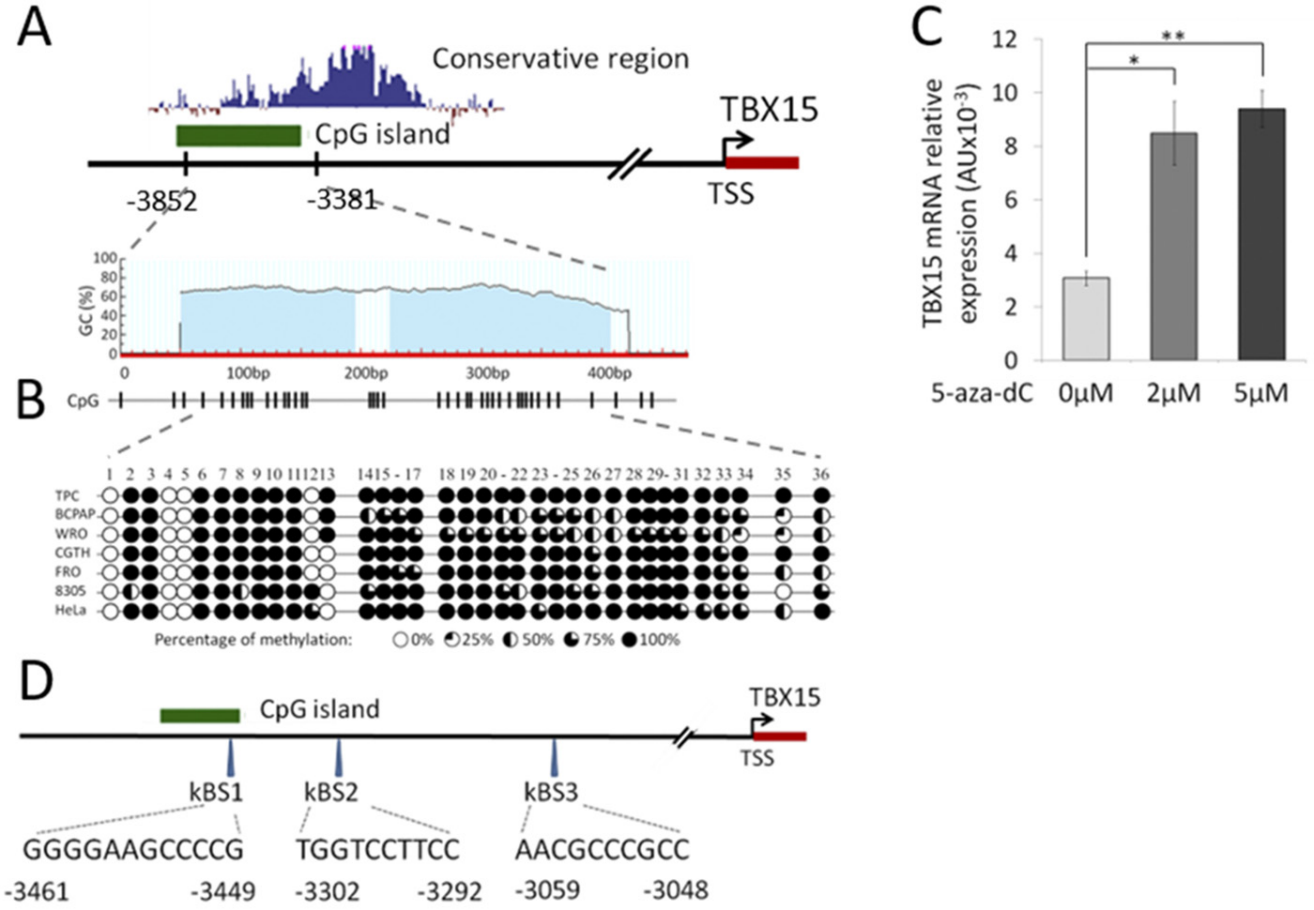
Methylation of the *TBX15* promoter in cancer cell lines. (A) Schematic diagram of *TBX15* promoter indicating the CpG island and the conservative area in the 5’flanking region. TSS: transcription start site. (B) Methylation of the studied CpG island in HeLa and six thyroid cancer cell lines. The bisulfite conversion and sequencing analysis cover 36 CpG sites in the studied CpG island. Each row represents the results for each cell line where the circles indicate the methylation status of each CpG site. (C) Treatment of TPC-1 cells with 5-aza-dC increased *TBX15*mRNA. qRT-PCR analysis and mRNA levels normalized to *RPL27*, showing the mean±SD of mRNA levels of two independent experiments in triplicates. * indicates p-value < 0.05 and ** p-value < 0.01. (D) Location of the *in silico* predicted NF-κB binding sites (kBS1, kBS2 and kBS3) in the studied region of the *TBX15* promoter.

The information about *TBX15* regulation is very scarce. To our knowledge there is only a study in the literature describing a CG rich sequence approximately 3 kb upstream of the *TBX15* transcription start point (TSS) with a possible role in *TBX15* expression. The study was carried out in human placentas showing that methylation of such CG rich sequence was altered in pathological placentas, and its methylation status was related to *TBX15* expression [13]. Databases pointed out that this region contains a CpG island and is highly conserved in vertebrates (UCSC Genome Browser), suggesting its functional importance. In Fig 1A we represented the conservative region in the 5’-distal position of *TBX15*. Hence, we hypothesized that *TBX15* regulation signals take place in that sequence, and then we explored such regulatory activity in human cancer cells.

### A 5’-distal conserved region of *TBX15* is highly methylated in cancer cells

First we investigated whether the level of methylation at the CpG island located 3 kb upstream of the *TBX15* TSS was related to *TBX15* transcription in cancer cells. Bisulphite sequencing of this CpG island, position -3852 bp to -3381bp of the *TBX15* gene, was performed in HeLa cells and six thyroid cancer cell lines (TPC-1, BCPAP, WRO, CGTH, FRO and 8305). As shown in Fig 1B, high levels of methylation along all CG sites analyzed were found in the seven cell lines (range 69%-89%); however, the levels of *TBX15* mRNA were much more different between these cell lines (S3 Table), being unfeasible to establish a correlation between methylation at the analyzed CpG island and transcription of *TBX15* in the studied cell lines. For example, in the cell line CGTH the *TBX15* mRNA expression was 22-fold higher than in TPC-1 (65x10^-3^ AU and 3.04x10^-3^ AU, respectively; S3 Table), but both cell lines presented similar methylation levels in the CpG island evaluated (Fig 1B). On the other hand, treatment of TPC-1 cells with the demethylating agent 5-aza-dC increased 2.6-fold the level of *TBX15* mRNA compared with untreated cells (Fig 1C), which would indicate a modulation of *TBX15* expression by epigenetic factors; most likely, other regulatory processes may also control the *TBX15* expression, as was indicated in human placentas [13].

Next, we searched for cis-regulatory elements in the upstream region of *TBX15* using four independent programs (see Materials and Methods). Three putative NF-κB binding sites were identified in the area containing the CpG island analyzed above, and are referred as kBS1, kBS2 and kBS3 in this study (Fig 1D). Other potential transcription factors binding sites were also predicted, but only by one or two of these programs. Based on these data and taking into account that NF-κB is an important factor in cancer, we considered NF-κB as a potentially relevant factor to regulate *TBX15* transcription.

### Activation of NF-κB by TNF-α increases *TBX15* mRNA expression in cancer cells

**Fig 2:**
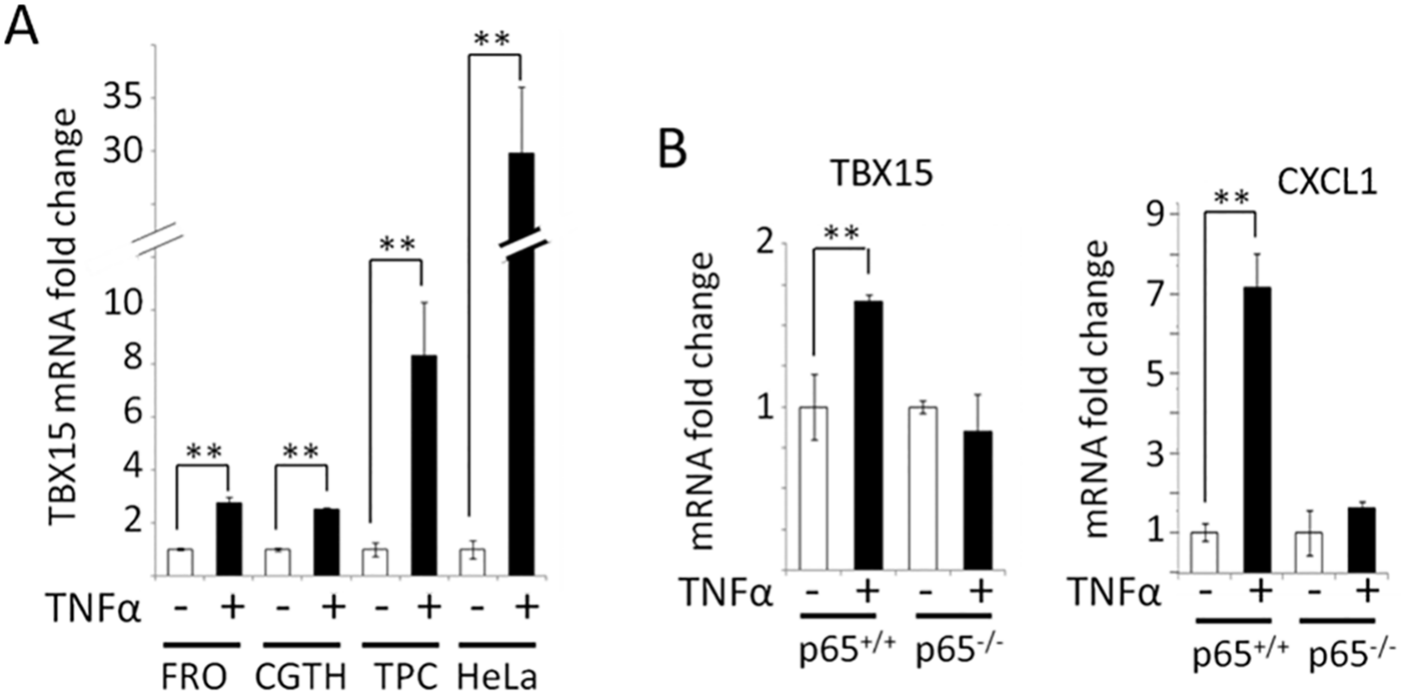
Expression of *TBX15*mRNA in different cell lines after stimulation with TNF-α. (A) qRT-PCR of *TBX15*mRNA analysis in FRO, CGTH, TPC-1 and HeLa cells after 2h of TNF-α treatment. (B) qRT-PCR of *TBX15*mRNA (left) and *CXCL1*mRNA (right) analysis in p65^+/+^ and p65^*-/-*^ MEF cells after TNF-α treatment. Data were normalized to *RPL27* and expressed as fold change referred to untreated cells whose *TBX15*mRNA or *CXCL1*mRNA relative expression was defined as 1. Data are mean±SD of mRNA levels of two independent experiments in triplicates. ** indicates p-value < 0.01.

To investigate if NF-κB was involved in the regulation of *TBX15* expression we stimulated HeLa cells and three thyroid cancer cell lines with TNF-α to activate NF-κB, and subsequently we measured the *TBX15* mRNA levels. As shown in Fig 2A, treatment of cells with TNF-α significantly increased the levels of *TBX15* mRNA, compared to untreated cells (increase ranging from 31.8- to 2.5-fold, depending of the cell line). The induction of *TBX15* expression by TNF-α was comparable to the induction of *CXCL1* expression measured in the same experiments, which was used as positive control for TNF-α activation of the NF-κB pathway (S1 Fig). To further confirm that *TBX15* expression was controlled by NF-κB activation, we analyzed the amount of *TBX15* mRNA in TNF-α stimulated p65^+/+^ and p65^-/-^ MEF cells, and we found that TNF-α increased the level of *TBX15* mRNA in p65^+/+^ but not in p65^-/-^ MEF cells (Fig 2B). In these experiments, activation of NF-κBp65 by TNF-α was verified by the induction of *CXCL1* expression in p65^+/+^ MEF cells after TNF-α stimulation. Thus, these data demonstrate that NF-κBp65 up-regulates *TBX15* transcription in MEF cells, and indicate that activated NF-κBp65 has a positive effect in *TBX15* regulation in human cancer cells.

### Effect of NF-κB inhibition by IκBα super-repressor in *TBX15* mRNA expression

**Fig 3:**
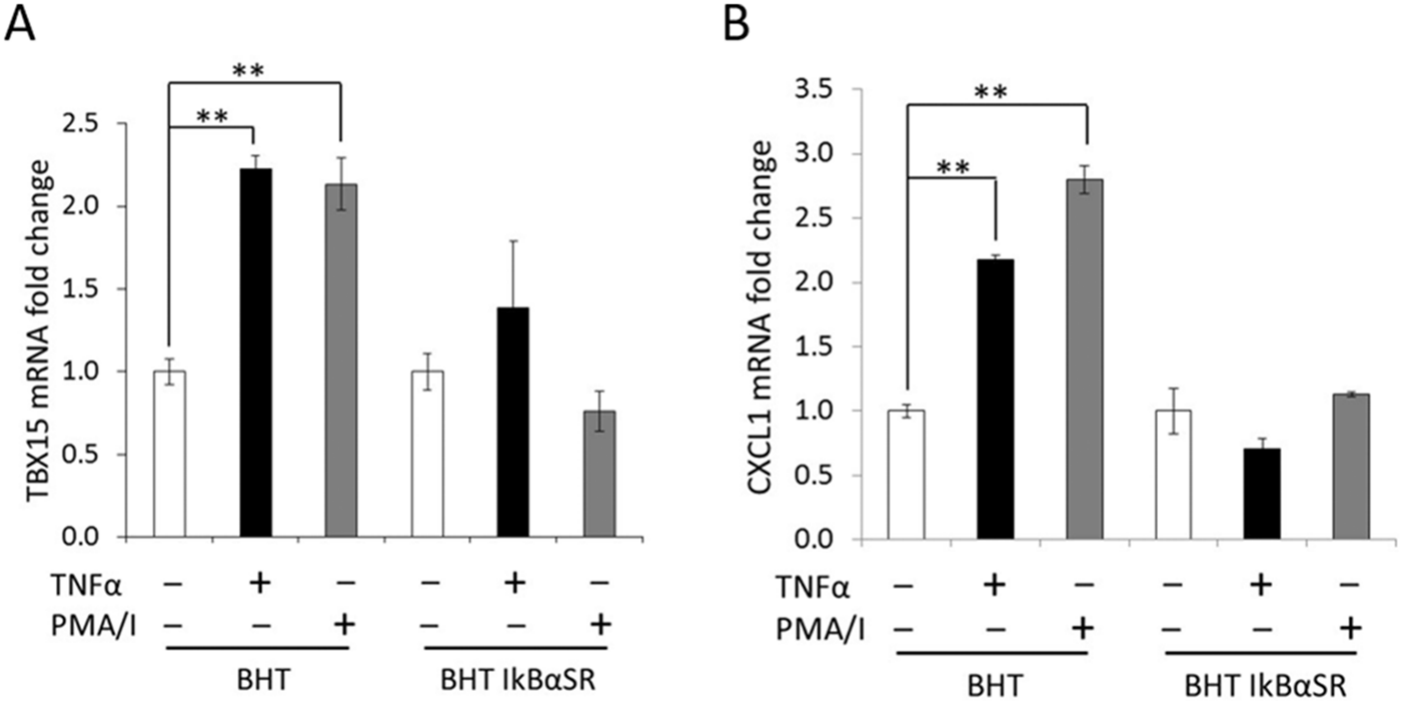
Expression of *TBX15*mRNA and *CXCL1*mRNA in BHT cell lines after stimulation with TNF-α and PMA/I. (A) qRT-PCR of *TBX15*mRNA analysis in BHT and BHT IkBαSR cells after 3h of TNF-α or PMA/I treatment. (B) qRT-PCR of *CXCL1*mRNA analysis in BHT and BHT IkBαSR cells after 3h of TNF-α or PMA/I treatment. Data were normalized to *RPL27* and expressed as fold change referred to untreated cells whose *TBX15*mRNA or *CXCL1*mRNA relative expression was defined as 1. Data are mean ± SD of mRNA levels in triplicates. ** indicates p-value < 0.01.

An additional evidence of the implication of NF-κB in *TBX15* regulation was provided using an IκBα super-repressor (IκBαSR) to inhibit NF-κB activation. Then, we analyzed the *TBX15* expression in BHT-101 and BHT-101 IκBαSR cells after TNF-α or PMA/I stimulation. BHT-101 IκBαSR cells constitutively express an IκBαRS sequestering NF-κB so is unable to response to activation stimuli. As shown in Fig 3A, after treatment with TNF-α or PMA/I, the levels of *TBX15* mRNA in BHT-101 IκBαSR cells were similar than in untreated cells. However, as expected, stimulation of BHT cells showed an increase of *TBX15* mRNA. In these experiments the expression of *CXCL1* was also evaluated and used as a positive control for activation of the NF-κB pathway, obtaining equivalent results than in the analysis of *TBX15* expression (Fig 3B). These results support the role of NF-κB canonical pathway in *TBX15* regulation in cancer cells.

### Identification of a *TBX15* regulatory region containing NF-κB binding sites

**Fig 4:**
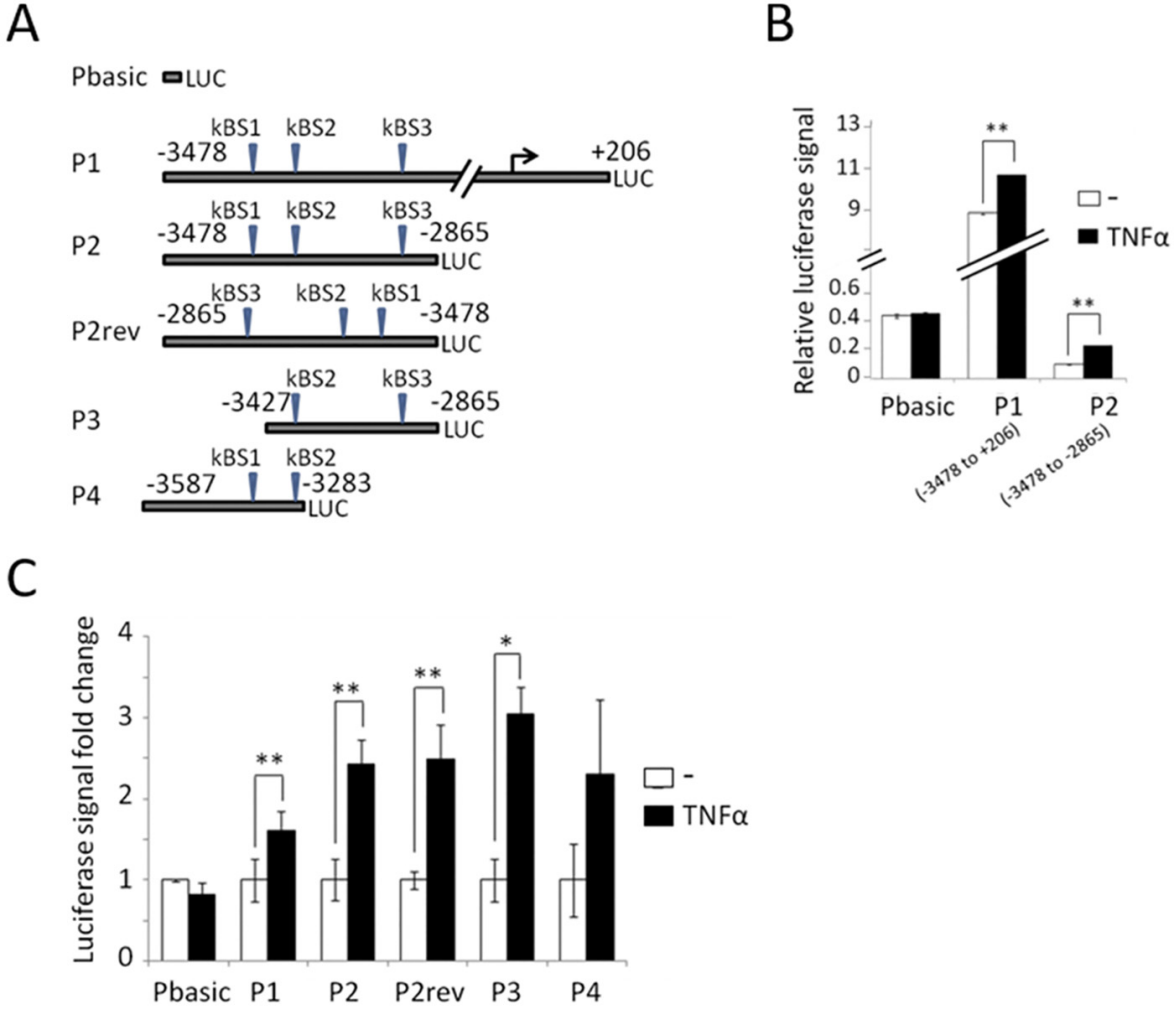
Functional analysis of the 5’-flanking region of the human *TBX15* gene. (A) Representation of the different luciferase reporter constructs used in the analysis, with predicted NF-κB binding sites (kBS1, kBS2 and kBS3). (B) The *TBX15* promoter and its 5’-flanking region activity measured by luciferase in TNF-α treated and untreated cells. Vector expressing the *Renilla* luciferase was co-transfected as an internal reference to correct the transfection efficiency. (C) Luciferase analysis of the different reporter constructions. Data was expressed as fold change referred to untreated cells as 1. At least three independent experiments were performed with duplicates. Bars represent the mean±SD. * indicates p-value < 0.05 and ** p-value < 0.01.

Luciferase reporter experiments were performed to study the *TBX15* promoter activity and to identify the regulatory region that responds to NF-κB. Five 5’-fragments of the human *TBX15* gene were cloned into the pGL4 luciferase reporter plasmid: a 3.68 kb fragment containing the three predicted NF-κB binding sites and the TSS (P1: -3478/+206) and four fragments from the 5’-flanking region of *TBX15* (P2: -3478/-2865, P2rev: -2865/-3478, P3: -3427/-2865 and P4: -3587/-3283) (Fig 4A). Following transfection of each construct in HEK293 cells, luciferase activity was evaluated in TNF-α -stimulated and non-stimulated cells and transfected cells with pGL4-basic were used as negative control. Analysis of luciferase activity in non-stimulated cells served to assess the promoter activity. As shown in Fig 4B, promoter activity was observed in the 3.68 kb P1 fragment of the *TBX15* promoter, but not in its 5’-flanking region represented by the P2 fragment. On the other hand, P1 and P2 fragments showed similar response to the endogenous activation of NF-κB by TNF-α (Fig 4B), and the response to the activated NF-κB was also observed in the rest of constructs, P2rev, P3 and P4 (Fig 4C). These results are in agreement with our indication before of activation of constitutive NF-κB regulating *TBX15* in different cell lines, and locate the regulatory region in the 5’-flanking position of the *TBX15* gene from -3478 to -2865. Since the P2 fragment contains the three predicted NF-κB binding sites in the 5’-distal region of *TBX15*, and because both P2 and P2rev showed similar response to NF-κB activation, we propose that the -3478/-2865 region of *TBX15* would integrate NF-κB signals in an orientation-independent manner to modulate positively the expression of *TBX15*.

**Fig 5:**
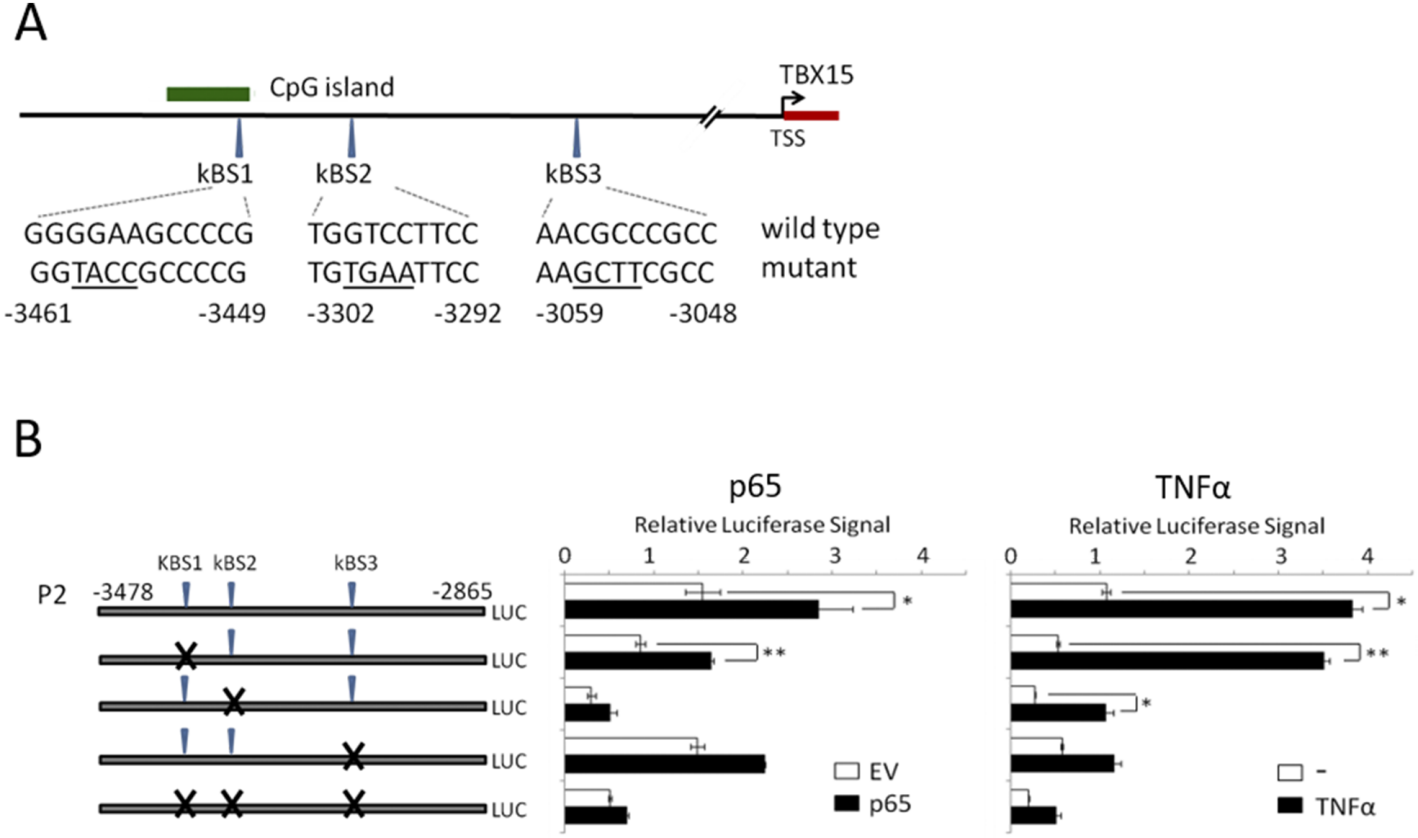
Identification of functional NF-κB responsive elements in the 5’-flanking region of the human *TBX15* gene. (A) Location and sequence of the predicted NF-κB binding sites (kBS1, kBS2 and kBS3) in the studied region of the *TBX15* promoter, with indication of altered sequence in mutated reporter constructs (underlined). Site directed mutagenesis was performed using P2 reporter construct to obtain the mutated reporter constructs. (B) Luciferase analysis of each mutated reporter constructs. *Left panel*, co-tansfected with the pCDNA3-p65 expression vector (p65) or the pCDNA3 empty vector (EV). *Right panel*, in nonstimulated or stimulated TNF-α cells. At least three independent experiments were performed with duplicates. Bars represent the mean±SD. * indicates p-value < 0.05 and ** p-value < 0.01.

The NF-κB responsive region of the 5’-flanking region of *TBX15* was further analyzed by introducing mutations in the P2 reporter construct to alter each of the predicted NF-κB binding site followed by luciferase assays. The mutated plasmids that were evaluated and their altered sequences are indicated in Fig 5A. The reporter assays performed in HEK293 cells along with a NF-κBp65 expression plasmid or a control empty vector, allowed identifying the NF-κBp65-responsive sites in the 5’-distal region of *TBX15*. The results are illustrated in Fig 5B. In agreement with our finding of NF-κBp65 up-regulating *TBX15* in MEF cells, cotransfection of the NF-κBp65 expression vector increased 2-fold the activity of the P2 wild type construct compared to the empty vector; however, no increase of activity was detected in the P2 triple mutant construct. The kBS1 mutant plasmid showed a similar increase of activity than the P2 wild type plasmid, after transfection with the NF-κBp65 expression vector. In contrast, the single kBS2 and kBS3 mutant plasmids were not activated by NF-κBp65, indicating that kBS2 and kBS3 sites are NF-κBp65 responsive elements. Furthermore, we observed that in the reporter assays with the control empty vector, the kBS2 mutant plasmid presented a reduced activity of >80% (Fig 5B), indicating the contribution of the kBS2 site to the basal expression of *TBX15*. Additional evidence of kBS2 and kBS3 as the NF-κB responsive elements in the 5’-flanking region of *TBX15* was provided by the luciferase reporter assays with the same P2 mutant constructs under TNF-α -stimulated conditions. Similar to ectopic NF-κBp65, activation of endogenous NF-κB with TNF-α increased the activity of the P2 wild type and the kBS1 mutant constructs, whereas much less response was observed with the kBS2 mutant construct and no response was observed with the kBS3 mutant construct (Fig 5B). It is interesting to point out that the activity of the P2 wild type plasmid after TNF-α stimulation was higher than with the ectopic expression of NF-κBp65 (4-fold and 2-fold, respectively, compared to control). Altogether, these results indicate that kBS2 and kBS3 are functional NF-κB motifs that respond to NF-κB increasing *TBX15* expression.

**Fig 6:**
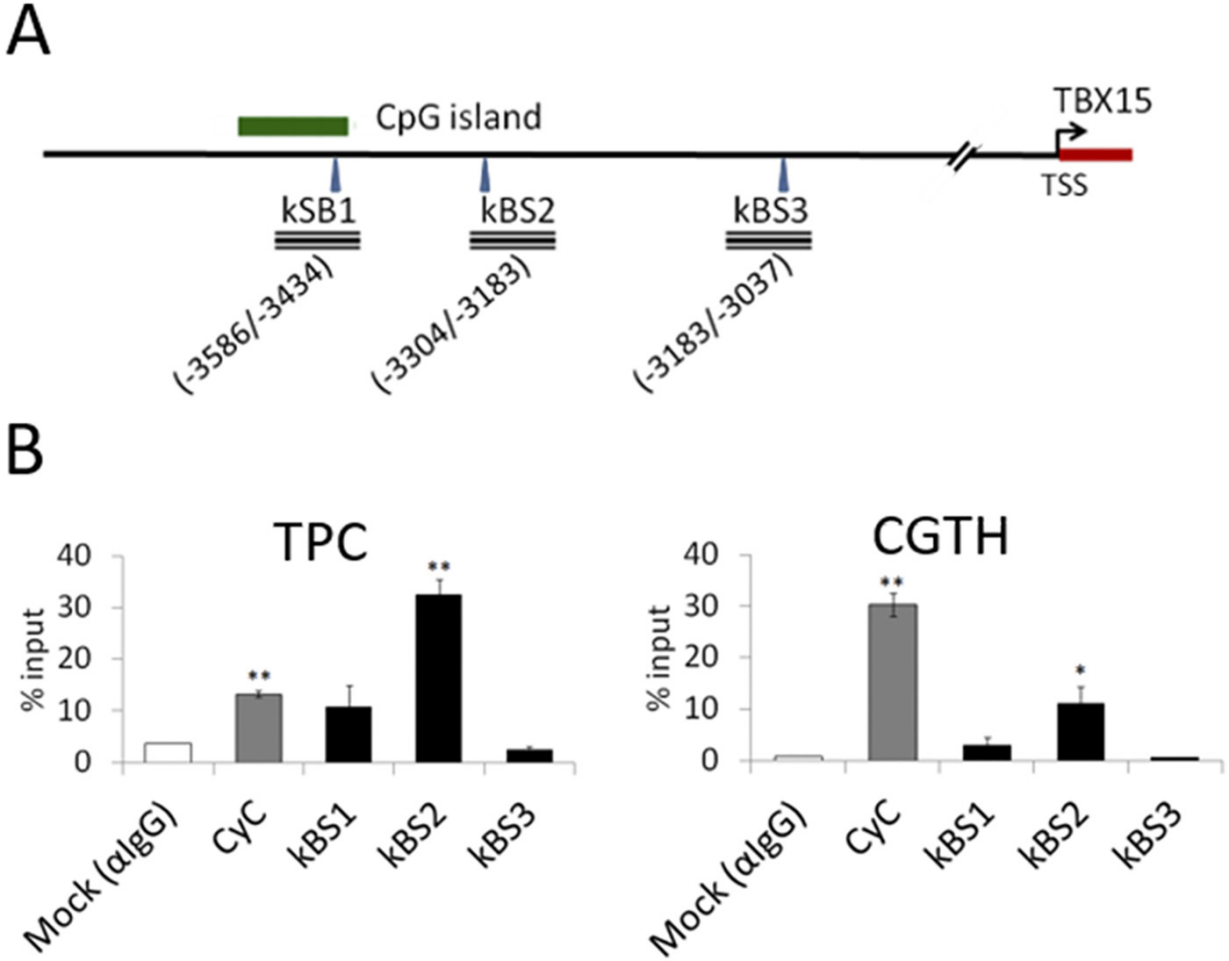
Chromatin inmunoprecipitation (ChIP) assays. (A) The PCR amplified regions comprising the predicted NFκB binding sites (kBS1, kBS2 and kBS3) are indicated as rectangles, with location of these specific DNA segments indicated by numbers. (B) TPC and CGTH cells were treated with TNF-α and ChIP assays performed using IgG (mock) and NFκBp65 antibodies. Imnmunoprecipitated DNA was analyzed by qPCR with primers targeted to the predicted NFκB binding sites (kBS1, kBS2 and kBS3). The quantitative data reflect occupancy calculated as % input and represented as mean±SD of two independent experiments. *: P<0.05; **: P<0.01.

Next, *in vivo* association of NF-κBp65 to the 5’-flanking region of *TBX15* was assessed by ChIP analysis in TPC and CGTH cells after NF-κB activation by TNF-α treatment (Fig 6). Using a NF-κBp65 antibody, the amplification with specific primer of the kBS1, kBS2 and kBS3 regions showed that NF-κBp65 binds to the kBS2 region, but not to the kBS1 or kBS3 (Fig 6B). These results, together with the analysis before of the lack of induction of *TBX15* expression in p65^-/-^ MEF cells, indicate that NF-κBp65 acts as a positive regulator of *TBX15* by direct interaction with a 5’-distal regulatory region of this gene.

## Discussion

*TBX15* is a T-box gene that encodes a transcription factor being crucial in development processes [1]. More recently, we have shown an antiapoptotic role of *TBX15*, together with its altered expression in thyroid cancer cells [15]. Others had also reported that *TBX15* controls the number of mesenchymal precursor cells and chondrocytes during development of the skeleton of the limbs [2]. These data indicate a role of *TBX15* in cell proliferation, and support the assumption of its implication in carcinogenesis, as it was previously indicated for some members of the T-box gene family [10, 11]. To understand the function of *TBX15* is crucial to disclose the mechanisms that regulate *TBX15*. In this study, we provide novel data showing that NF-κB signaling pathway upregulates *TBX15* expression in cancer cells.

To study *TBX15* regulation, we center our attention in a 5’-conserved region of *TBX15* that showed regulatory functions in placentas [13]. This region contains three putative NF-κB responsive elements as revealed by our *in silico* sequence analysis (Fig 1D). Of importance, we found that *TBX15* mRNA expression was increased in response to TNF-α in HeLa and three thyroid cancer cell lines; moreover, the analysis of the *TBX15* expression in p65^-/-^ MEF cells stimulated with TNF-α indicated that the effect was mediated by NF-κB. Further evidences of a role of NF-κB in *TBX15* regulation were given by using BHT-101 IκBαSR cells that stably expressed an IκBα super-repressor that inhibits NF-κB. These cells when stimulated with TNF-α or PMA/I presented basal levels of *TBX15* mRNA, which was in contrast with the induction of *TBX15* mRNA found in stimulated BHT-101, indicating that NF-κB signaling was required to induce *TBX15* expression. We found that a fragment of 600 bp in the 5’-flanking region of *TBX15* that containing the three predicted NF-κB binding sites had regulatory activity, responding to TNF-α or to ectopic expression of NF-κBp65. Functional analysis of this region using reporter constructs with mutations in the three putative NF-κB binding sites showed that two of the three sites, located on -3302 and -3059 of the *TBX15* gene (kBS2 and kBS3, respectively; Fig 5A), were functional. We demonstrated that the common NF-κB subunit p65 is important for the positive response of these motifs to TNF-α. In addition, ChIP assays indicated a direct interaction of NF-κBp65 with the kBS2 binding site in TPC1 and CGTH cells. Here, we propose that a cooperative action of the two functional NF-κB motifs takes place to upregulate *TBX15*. The fact that reporter assays showing higher activity after stimulation with TNF-α than with NF-κBp65, also suggest that, besides p65, other factors activated by TNF-α including different NF-κB subunits would recognize these functional motifs. In this context it is feasible to assume that the two functional NF-κB motifs identified in this study integrate the NF-κB signals required to positively modulate the transcription of *TBX15*. Most likely, additional factors would be needed to control *TBX15* transcription acting in a cell type-dependent manner. Thus, in pathological placentas, *TBX15* is downregulated by PDX1 in a methylation-dependent manner [13]; and differential *TBX15* expression was found in adipocytes [9]. In the present study we show that demethylation with 5-aza-dC resulted in an increase of the *TBX15* mRNA expression in TPC-1 thyroid cancer cells; however, no correlation was found between methylation levels in the CpG island of *TBX15* analyzed and the *TBX15* mRNA expression, in nine thyroid cancer cell lines and HeLa cells. We cannot discard a possible association between the methylation status of other CG sites along the *TBX15* promoter and the expression of *TBX15*. Of interest, methylation status of the *TBX15* promoter related to clinicopathological features of prostate cancer was reported, although the study did not mention *TBX15* expression [14]. Therefore, at present, the significance of methylation in the regulation of *TBX15* needs to be investigated. Nevertheless, it seems that *TBX15* undergoes up- or down-regulation depending on the cell type and conditions, with expected effects on its target genes. More studies are needed to determine the genetic and epigenetic mechanisms of *TBX15* regulation.

A relevant issue in the present study is the link between *TBX15* and NF-κB, and this novel discovery would be important in development and cancer processes. NF-κB plays a central role in many cellular processes via the regulation of its target genes [20, 21]. Also, the activation of NF-κB in many types of cancer has been extensively reported, including thyroid cancer [22, 23]; and, the effects of NF-κB in cancer are related to the activation of cell proliferation and inhibition of apoptosis [24, 25]. Interestingly, we have also reported an antiapoptotic function of *TBX15* [15] that together with the present study indicates that, by activating *TBX15*, NF-κB may promote the antiapoptotic activity of *TBX15*, which points out *TBX15* as a possible downstream gene in the antiapoptotic activity of NF-κB. The expression of *TBX15* in cancer is unknown, but because NF-κB modulated the expression of *TBX15* we speculate that alterations of *TBX15* expression would be expected in different types of cancer with consequences in cancer development. To this regard, *TBX15* expression is altered in thyroid cancer cell lines ([15], and S3 Table) and the activation of NF-κB in cancer cells and tumor of the thyroid is well known [26, 27].

In development processes, there are some hints that also invite us to think of *TBX15* as a possible downstream target of NF-κB. During embryonic limb formation, NF-κB acts to transmit growth factor signals between the ectoderm and the mesenchyme [28], and *TBX15* controls the number of mesenchymal precursor cells [2]; therefore, *TBX15* could be a downstream factor of the ectoderm and the mesenchyme communication mediated by NF-κB.

In conclusion, here we have demonstrated the NF-κB mediated upregulation of *TBX15* in cancer cells, identifying two NF-κB functional motifs in the 5’-flanking region of *TBX15*. This novel finding together with our previous discovery of an antiapoptotic function of *TBX15*, support the implication of *TBX15* in carcinogenesis. Here we also propose a link between *TBX15* and NF-κB. So, further investigation is warranted to understand the role of *TBX15* in cancer and development processes, and in relation to NF-κB.

## Supporting Information

S1 Fig Expression of *CXCL1*mRNA in FRO, CGTH, TPC-1 and HeLa cells after stimulation with TNF-α. Data were normalized to *RPL27* and expressed as fold change referred to untreated cells whose *CXCL1*mRNA relative expression was defined as 1. Data are mean ± SD of mRNA levels of two independent experiments in triplicates. ** indicates p-value < 0.01.
(DOCX)

S1 Table Primer sequences used in this study. (DOCX)

S2 Table Composition of the solutions used in ChIP analysis. For luciferase constructions, restriction enzymes sites are in bold. For point mutation construction primers, changed nucleotides are marked in bold letter. BS-PCR: bisulfite polymerase chain reaction. Ta: annealing temperature.
(DOCX)

S3 Table *TBX15* mRNA relative quantification in cell lines. Data are normalized to the reference gene *RPL27* and expressed as mean ± SD.
(DOCX)
