# Supplementary material for: NF-κB Mediates the Expression of TBX15 in Cancer Cells
Source: PLoS One. 2016 Jun 21;11(6):e0157761. doi: 10.1371/journal.pone.0157761 (PMC4915632; doi:10.1371/journal.pone.0157761)
Supplement: S1 Fig — Data were normalized to RPL27 and expressed as fold change referred to untreated cells whose CXCL1mRNA relative expression was defined as 1. Data are mean ± SD of mRNA levels of two independent experiments in triplicates. ** indicates p-value < 0.01. (DOCX) [file pone.0157761.s001.docx]

**S1 Fig**


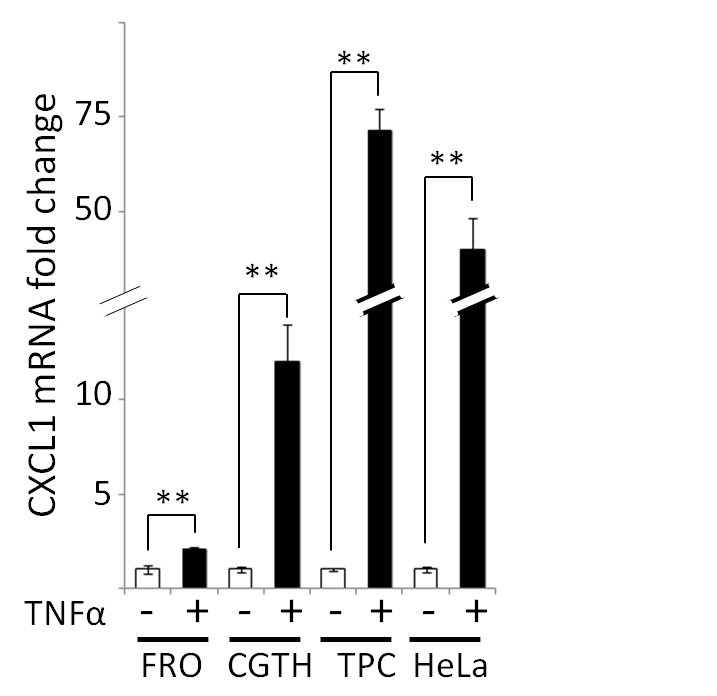


**S1 Fig**. Expression of *CXCL1*mRNA in FRO, CGTH, TPC-1 and HeLa cells after stimulation with TNF-α. Data were normalized to *RPL27* and expressed as fold change referred to untreated cells whose *CXCL1*mRNA relative expression was defined as 1. Data are mean ± SD of mRNA levels of two independent experiments in triplicates. ** indicates p-value < 0.01.
