# Supplementary material for: NF-κB Mediates the Expression of TBX15 in Cancer Cells
Source: PLoS One. 2016 Jun 21;11(6):e0157761. doi: 10.1371/journal.pone.0157761 (PMC4915632; doi:10.1371/journal.pone.0157761)
Supplement: S1 Table — (DOCX) [file pone.0157761.s002.docx]

**S1 Table**. Primer sequences used in this study.
