# Supplementary material for: NF-κB Mediates the Expression of TBX15 in Cancer Cells
Source: PLoS One. 2016 Jun 21;11(6):e0157761. doi: 10.1371/journal.pone.0157761 (PMC4915632; doi:10.1371/journal.pone.0157761)
Supplement: S2 Table — For luciferase constructions, restriction enzymes sites are in bold. For point mutation construction primers, changed nucleotides are marked in bold letter. BS-PCR: bisulfite polymerase chain reaction. Ta: annealing temperature. (DOCX) [file pone.0157761.s003.docx]

| **S2 Table**. Composition of the solutions used in ChIP analysis. | |
| --- | --- |
| **Solution Name** | **Composition** |
| PBS | phosphate buffered saline |
| PBS/Mg | PBS containing 1mM MgCl_2_ pH 8.0 |
| L1 buffer | 50mM Tris pH 8.0, 10mM EDTA, 0.1% IGEPAL 630, 10% glycerol, 1mM DTT. Protease inhibitor cocktail tablets (*cOmplete*, Roche) were added. |
| SDS lysis buffer | 1% SDS, 50mM Tris pH 8.0, 10mM EDTA |
| Low ionic strengh dilution buffer | 50mM NaCl, 10mM HEPES pH 7.4, 1% IGEPAL 630, 10% glycerol, 1 mM DTT. Protease inhibitor cocktail tablets (cOmplete, Roche) were added. |
| High salt wash buffer | 500mM NaCl, 0.1% SDS, 1% IGEPAL 630, 2mM EDTA, 20mM Tris pH 8.0 |
| LiCl wash buffer | 0.25M LiCl, 1% IGEPAL 630, 1% deoxycholate, 1mM EDTA, 10mM Tris pH 8.0 |
| TE | 10mM Tris pH 8.0, 1mM EDTA |
| Elution buffer | 1% SDS, 0.1M NaHCO_3_ |
|  |  |
