# Supplementary material for: NF-κB Mediates the Expression of TBX15 in Cancer Cells
Source: PLoS One. 2016 Jun 21;11(6):e0157761. doi: 10.1371/journal.pone.0157761 (PMC4915632; doi:10.1371/journal.pone.0157761)
Supplement: S3 Table — Data are normalized to the reference gene RPL27 and expressed as mean ± SD. (DOCX) [file pone.0157761.s004.docx]

**S3 Table**. *TBX15* mRNA relative quantification in cell lines. Data are normalized to the reference gene *RPL27* and expressed as mean ± SD

| *TBX15* mRNA relative quantification (x10^-3^) | |
| --- | --- |
| SS1N | 46.5 ± 2.05 |
| TPC | 3.04 ± 0.40 |
| BCPAP | 14.60 ± 2.01 |
| WRO | 36.20 ± 8.25 |
| CGTH | 65.00 ± 14.30 |
| FRO | 2.00 ± 0.31 |
| 8305 | 3.70 ± 0.20 |
| HeLa | 37.50 ± 3.19 |
